# The correlation between posttraumatic growth and social support in people with breast cancer: A meta-analysis

**DOI:** 10.3389/fpsyg.2022.1060150

**Published:** 2022-12-15

**Authors:** Xiaojing Ma, Xiao Wan, Chaoran Chen

**Affiliations:** Institute of Nursing and Health, College of Nursing and Health, Henan University, Kaifeng, Henan, China

**Keywords:** breast cancer, posttraumatic growth, social support, meta-analysis, review

## Abstract

Posttraumatic growth (PTG) is consistently reported to be associated with social support among people with breast cancer. But so far there is no consensus on the size and direction to which social support are related to PTG in people with breast cancer. Thus, a meta-analysis was performed by us to quantitatively synthesize the previous results. This meta-analysis followed the PRISMA 2020 guidelines. We searched PubMed, PsycINFO, Web of Science, Embase, Chongqing VIP Information Co., Ltd. (VIP), China National Knowledge Infrastructure (CNKI), and WANFANG DATA databases prior to 1 June 2022. A random effects model of Stata software (version 17.0) was employed to compute the pooled association coefficient and examine a series of moderating factors: economic level, publication type, region, year of publication, participants’ age, and social support measurement tools. Ultimately, 31 studies including 6,380 breast cancer patients were identified. This meta-analysis offers evidence of a highly positive correlation between PTG and social support among people with breast cancer (*r* = 0.425). Economic level, region, and social support measurement tools moderated the link between PTG and social support among people with breast cancer. Whether variables such as disease stage, time since diagnosis, and disease treatment moderate the link between PTG and social support among people with breast cancer can be further investigated in the future.

## Introduction

Breast cancer is one of the most common cancers affecting women worldwide (Xu et al., 2022). Based on global cancer statistics, breast cancer exceeded lung cancer to become the most common cancer in 2020, with approximately 2.26 million new cases and 680,000 deaths (Sung et al., 2021). Although the global five-year survival rate of people with breast cancer after diagnosis is >70% (Maajani et al., 2019), the diagnosis and treatment of breast cancer still have a strong negative impact on people’s mental health and trigger various negative psychological responses, such as depression (Denieffe et al., 2014), anxiety (Schmid-Büchi et al., 2011), fear of recurrence (Soriano et al., 2021), and posttraumatic stress disorder (Koutrouli et al., 2012), etc. However, some studies have found that as their cancer progresses, cancer patients often experience positive psychological changes, which are called to posttraumatic growth (PTG; Liu, 2020; Baník et al., 2022).

The definition of PTG is the positive psychological changes that an individual perceives in his or her fight against a traumatic incident (Tedeschi and Calhoun, 2004). Scholars found that differing from reactions to slight or daily pressure and the process of people growth and development, PTG refers to personal efforts to manage the influence of trauma on his or her life and try to deal with their experiences and ramifications (Linley and Joseph, 2004; Tedeschi and Calhoun, 2004; Chen et al., 2021). It is usually evaluated with the Posttraumatic Growth Inventory (PTGI) complied by Tedeschi and Calhoun (1996). The PTGI involves five dimensions: personal strength, relating to others, appreciation of life, new possibilities, and spiritual change and consists of 21 items scored by the Likert. 6-point scoring method, with high scores suggesting positive growth. Studies have found that numerous people with breast cancer have experienced PTG (İnan and Üstün, 2014; Paredes and Pereira, 2018; Li et al., 2019; Karimzadeh et al., 2021), and the study result of Bourdon et al. (2019) found that the PTG level of people with breast cancer was higher than that of healthy people.

In the past few years, scholars worldwide have actively explored the factors that influence PTG in breast cancer patients and found that social support is one of the psychosocial elements that is beneficial to the experience of PTG (Casellas-Grau et al., 2016; Hasson-Ohayon et al., 2016; Shen et al., 2016; Li, 2017). Although there is no single definition of social support, it usually refers to the support behaviors that individuals obtain from other individuals and social networks (Heller et al., 1986). The diverse choices of social support assessment tools are caused by differences in research perspectives, But there are several commonly used social support measurement tools. From the perspective of individual subjective feelings, Zimet et al. (1988) compiled the Multidimensional Scale of Perceived Social Support (MSPSS), which gauges perception of friends, family, and significant others’ support and includes 12 items scored on a Likert 7-point scale. The higher score, the stronger the sense of social support. Jiang (1999) translated and revised the MSPSS into the Chinese version of Perceived Social Support (PSSS). Xiao (1994) regarded social support as a combination of subjective sense and objective means and he developed the Social Support Rating Scale (SSRS), which is composed of 10 items and includes the three dimensions of subjective support, objective support, and social support utilization.

Many scholars have checked up the link between PTG and social support among breast cancer patients; however, the results are mixed. Some researchers have found a highly positive connection between PTG and social support (*r* = 0.470, 0.736, 0.574; Ma, 2014; Hasson-Ohayon et al., 2016; Li, 2018), some research have discovered a moderate connection between PTG and social support (*r* = 0.349, 0.360, 0.370; Tong et al., 2013; Aflakseir et al., 2018; He et al., 2018), while some study results have shown a weak relation between PTG and social support (*r* = 0.210, 0.123; Cohen and Numa, 2011; Liu, 2019). Some researchers have even found no significant connection between PTG and social support (Cordova et al., 2001; Kroemeke et al., 2017; Zhang et al., 2017). Thus, the first purpose of the study was to integrate previous empirical studies on the correlation between PTG and social support to assess the direction and size of the correlation between the two factors and provide evidence for whether social support is related to PTG.

We checked if the link between PTG and social support among people with breast cancer in previous studies might be due to the influence of potential moderators such as economic level, region, year of publication, participants’ age, publication type, and social support measurement tools. First, Bozo et al. (2009) and Wang (2014) found a strong link between PTG and social support among breast cancer patients in developing countries (*r* = 0.420, 0.631), while Cohen and Numa (2011) and Romeo et al. (2019) found a weak connection between PTG and social support among breast cancer patients in in developed countries (*r* = 0.210, 0.244). Thus, the connection between PTG and social support may vary depending on the economic level. Second, compared to other countries, China has a unique system and cultural background. Therefore, the connection between PTG and social support may vary according to the region. Third, the incidence and mortality of breast cancer are rising yearly, and breast cancer has become the most common cancer in the world (Sung et al., 2021). Hence, the link between PTG and social support among people with breast cancer may also change over time. Fourth, the research results of Karlsen et al. (2016) and Yeo et al. (2020) both showed that compared with older breast cancer survivors, young survivors are more affected by cancer, have greater emotional distress and worse psychological adjustment. Therefore, the connection between PTG and social support among people with breast cancer may differ. Fifth, Sterne et al. (2000) found that in general, research with significant results is easier to publish, leading some scholars to overstate the true relationship between variables. Hence, this study involved the dissertations which were not formally published in journals. The articles were divided by us into journals and dissertations according to publication type. At the same time, we tested whether publication type would adjust the connection between PTG and social support. Finally, considering the measurement of social support, the characteristics of some measurement tools are different. For example, the SSRS centers on the measurement of objective support and the extent of support use (Xiao, 1994), while the MSPSS and the PSSS emphasize the initiative of individuals in social support (Zimet et al., 1988; Jiang, 1999). The PSSS is the MSPSS after sinicization. Hence, social support measurement instruments may moderate the correlation between PTG and social support among people with breast cancer.

In summary, this study carried on a meta-analysis of the connection between PTG and social support among people with breast cancer, investigated the direction and size to which social support has a bearing on PTG among people with breast cancer, and checked whether the connection between social support and PTG is adjusted by (a) economic level, (b) region, (c) publication type, (d) year of publication, (e) participants’ age, and (f) social support measurement tools.

## Materials and methods

We registered the protocol of this meta-analysis in PROSPERO CRD42022311520. The meta-analysis abided by the PRISMA 2020 guidelines (Page et al., 2021), for searching articles, extracting results and describing the systematic processes.

### Literature search

The following seven databases were searched by us for research on the link between PTG and social support among people with breast cancer published from inception to 1 June 2022: PsycINFO, PubMed, Web of Science, Embase, Chongqing VIP Information Co., Ltd. (VIP), China National Knowledge Infrastructure (CNKI), and WANFANG DATA. For the Chinese databases, the search terms included “breast cancer” OR “breast tumor” AND “posttraumatic growth” OR “benefit finding” OR “stress-related growth” AND “social support.” For the English databases, See PubMed’s detailed search strategy for Supplementary material. We also manually checked the reference list of retrieved articles to find potential relevant research.

### Study selection criteria

The literature records were independently screened by two reviewers for possibly eligible articles. The inclusion criteria of articles were as followed: (1) patients were diagnosed with breast cancer by histopathology; (2) the PTGI or a revised PTGI scale were used to measure PTG; (3) there was no restriction on the social support scale; (4) the Pearson’s association coefficient *r* or *t* and *β* values that could be changed to *r* values were reported in articles; (5) when the data from dissertations, conference papers and journal articles came from the same dataset, we used the one published in the journal. However, if the journal article did not involve the complete dataset, we used the original dissertation with an analysis of the full dataset.

The exclusion criteria were (1) conference reports; (2) low-quality research; (3) articles not written in Chinese or English; and (4) research with obvious data mistakes.

### Quality assessment

Two reviewers independently used the 9-item Joanna Briggs Institution Critical Appraisal Checklist to assess the quality of methods used in all studies (Munn et al., 2015; see Supplementary material for detailed items). The answer options for every item were “Yes,” “no,” “not applicable,” and “unclear.” 1 point for “Yes,” 0 point for “no,” “unclear,” and “not applicable.” The higher the score, the higher the quality of the article. We solved the questions or disagreements arising from the article quality evaluation process by concentrated discussion or seeking advice from third-party specialists. As the final article quality scores were ≥6 (Table 1), we believed that the quality of the research included is good.

**Table 1:** Characteristics of the 31 studies involved in this meta-analysis.

Study author (year)	Country	Publication type	Age/rank^a^	*r*	N	Social support measurement	JBI score
			mean ± SD				
Cordova et al. (2001)	United States	Journal	54.7 ± 12.1	0.130	70	DUKE-SSQ	7
Bozo et al. (2009)	Turkey	Journal	46.28 ± 9.23	0.420	104	MSPSS	7
Cohen and Numa (2011)	Israel	Journal	59.26 ± 10.01	0.210	124	MSPSS	7
Tong et al. (2013)	China	Journal	45.19 ± 2.54	0.349	169	PSSS^b^	9
Lu (2014)	China	Journal	50.47 ± 9.51	0.239	159	PSSS^c^	8
Ma (2014)	China	Dissertation	49.87 ± 10.03	0.736	300	PSSS^c^	8
Wang (2014)	China	Journal	N/A	0.631	138	PSSS^c^	7
Dou et al. (2016)	China	Journal	N/A	0.736	280	PSSS^c^	7
Hasson-Ohayon et al. (2016)	Israel	Journal	53.24 ± 9.24	0.470	80	CPASS	7
Shen et al. (2016)	China	Journal	49.87 ± 10.03	0.736	300	PSSS^c^	7
Chen et al. (2017)	China	Journal	42.98 ± 4.29	0.399	224	SSRS	8
Gao and Shi (2017)	China	Journal	50.88 ± 9.2	0.318	102	PSSS^c^	7
Kroemeke et al. (2017)	Poland	Journal	62.27 ± 8.38	0.060	84	SSE-Q	6
Li (2017)	China	Journal	52.24 ± 8.17	0.491	295	PSSS^c^	8
Qiu and Zhou (2017)	China	Journal	43.70 ± 7.94	0.269	83	MSPSS	6
Tomita et al. (2017)	Japan	Journal	59.08 ± 10.06	0.290	157	JMS-SSS	6
Zhang et al. (2017)	China	Journal	52 ± N/A	0.056	210	PSSS^b^	8
Aflakseir et al. (2018)	Iran	Journal	52 ± 12.32	0.370	196	MSPSS	9
He et al. (2018)	China	Journal	48.86 ± 10.06	0.360	160	SSRS	9
Li (2018)	China	Dissertation	N/A	0.574	424	MSPSS	7
Liu (2018)	China	Dissertation	48.78 ± 7.56	0.504	325	PSSS^c^	7
Mao (2018)	China	Dissertation	49.07 ± 7.67	0.515	193	PSSS^c^	8
Yeung and Lu (2018)	United States	Journal	54.7 ± 8.61	0.440	118	MOS-SS	7
Liu (2019)	China	Dissertation	46.88 ± 13.09	0.123	612	FBNRI	8
Romeo et al. (2019)	Italy	Journal	54.3 ± 8.0	0.244	123	MSPSS	7
Yan et al. (2019)	China	Journal	49.91 ± 11.73	0.569	180	SSRS	9
Liu et al. (2020)	China	Dissertation	28–68	0.453	112	SSRS	8
Liu et al. (2021)	China	Journal	48.21 ± 3.16	0.284	174	SSRS	8
Zhang (2021)	China	Dissertation	47 ± 0.95	0.481	236	SSRS	9
Zhou (2021)	China	Journal	N/A	0.668	358	PSSS^c^	8
Du et al. (2022)	China	Journal	20–40	0.294	290	SSRS	8

a N/A, Not reported.

b PSSS compiled by Blumenthal.

c The Chinese version of PSSS revised by Jiang.

DUKE-SSQ, Duke—UNC Functional Social Support Questionnaire; MSPSS, Multidimensional Scale of Perceived Social Support; PSSS, Perceived Social Support Scale; CPASS, Cancer Perceived Agents of Social Support; SSRS, Social Support Rating Scale; SSE-Q, Social Support Effectiveness Questionnaire; JMS-SSS, Jichi Medical School Social Support Scale; MOS-SS, Medical Outcomes Study Social Support Survey; FBNRI, Furman and Buhrmester Net-work of Relationships Inventory.

### Data extraction

Two researchers independently used a purpose-designed form to extract data, and disagreements arising from the extraction process were addressed by discussion. The collected research is encoded by us with the following traits: study information, country, publication year, participant characteristics, publication type, sample size, correlation coefficients between PTG and social support, and social support measurement tools. If the research did not inform the correlation coefficient *r*, but informed *t* and *β* values, it should be changed to *r* value according to the following corresponding formula: *r* = $\sqrt{\frac{t^{2}}{t^{2}+df}}$ , *r* = *β* × 0.98–0.05 (*β* < 0) [−0.5 < *β* < 0.5] *r* = *β* × 0.98 + 0.05 (*β* ≥ 0)(Card, 2012). Furthermore, if multiple effect sizes of PTG and social support obtained in identical samples, we only selected the overall effect size.

### Statistical analysis

We used the inverse variance method to calculate the pooled association coefficients and their corresponding 95% confidence intervals (CIs) between PTG and social support (Moles, 2009). Specifically, we used Fisher transform to convert *r* value to corresponding Fisher Z value, weighted according to the sample size with 95% CIs: Z = 0.5*ln[(1 + r)/(1 − r)]. Meanwhile, V_Z_ = 1/n − 3 is the variance of Z, and SE_Z_ = $\;\sqrt{\left(1/n-3\right)}$ is the standard deviation of Z. According to the suggestions by Lipsey and Wilson (2001), low, moderate, and high correlations correspond to effect size *r* values of 0.10, 0.25, and 0.40, respectively. A random effect model was used by us to conduct data analysis. Compared with the fixed effect model, the random effect model more suits for the current meta-analysis because the size of the common potential effects of all research in this meta-analysis are not assumed (Borenstein et al., 2021; Huang et al., 2022). Moreover, we used Cochran’s Q and *I*^2^ statistics to appraise the heterogeneity across studies (Higgins et al., 2003). Heterogeneity between studies had statistical significance when *p* < 0.05 or *I*^2^ > 75%.

Potential moderation effects were suggested by a large level of heterogeneity. Meta-regression analysis was employed by us to check whether the result of the continuous moderating variable was significant. Subgroup analysis was used by us to examine whether the result of categorical moderating variable was significant. Furthermore, to appraise the effect of single study on the summary association coefficients and to examine the steadiness of the correlation between PTG and social support, we conducted a sensitivity analysis. Funnel plots were applied to detect potential publication bias. In addition, we performed Egger’s linear regression test to assess publication bias (Egger, 1997). We used Stata software (version 17.0) to conduct all statistical analyses.

## Results

### Study characteristics

**Figure 1:**
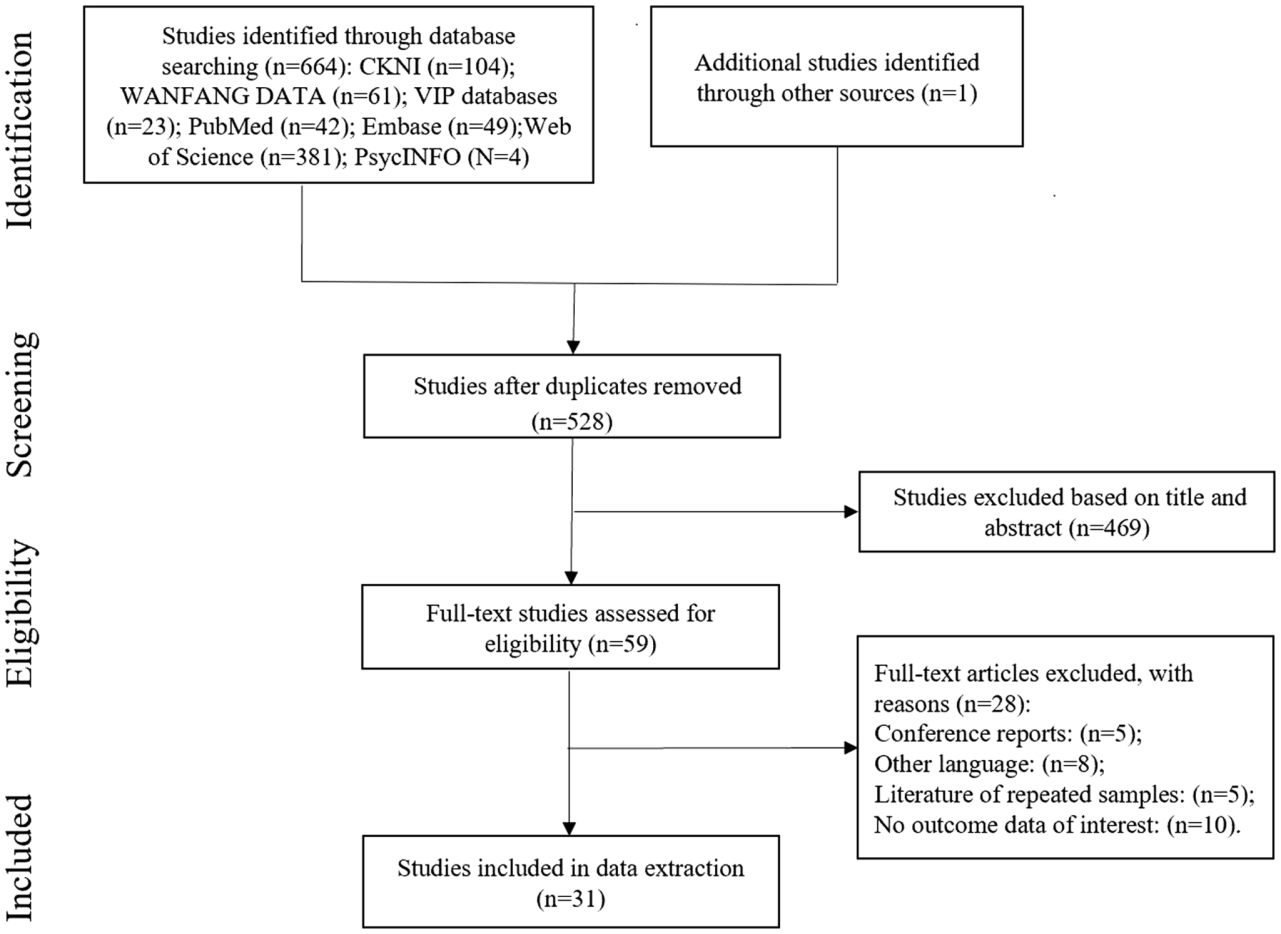
Flow chart of the research selection process.

528 studies without duplicates were identified by our search strategy (see Figure 1 for the flow chart of the research selection process). We conducted a qualification examination on the full text of 59 articles after reading the titles and abstracts. Among them, we excluded 28 studies since they were conference reports (*n* = 5), were in other languages (*n* = 8), duplicate samples (*n* = 5), or had no interesting result data (*n* = 10). Ultimately, 31 studies were included and the total sample size is 6,380 patients, all from articles published after 2001. Table 1 summarizes the features of the included studies. The survey sample size ranged from 70 to 612 participants. Among the 31 studies, two were from the United States and Israel each, 22 were from China, and five were from Turkey, Poland, Japan, Iran, and Italy each.

### Pooled analyses

**Figure 2:**
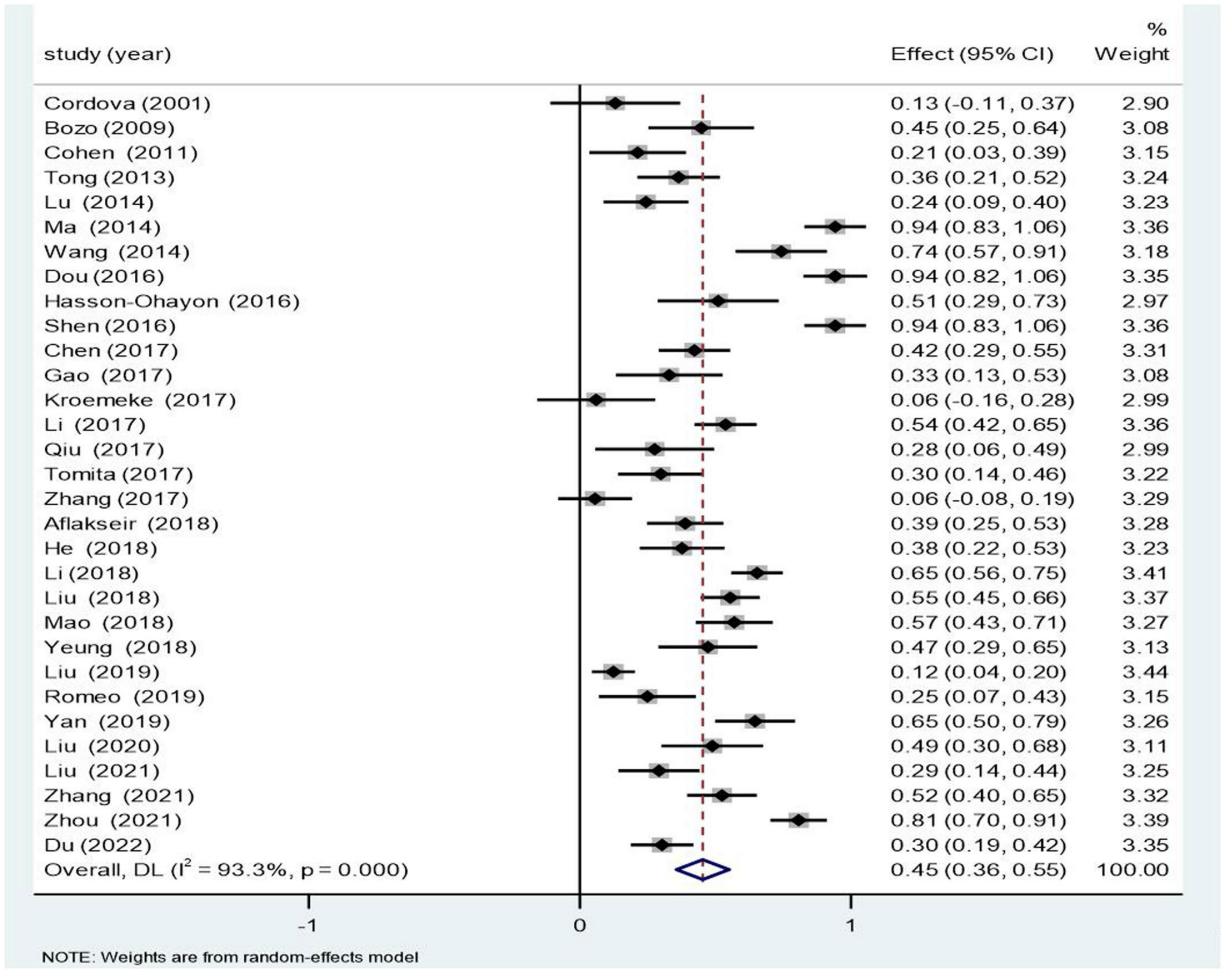
Forest plots for the link between posttraumatic growth and social support.

As demonstrated in Figure 2 and Table 2, the random-effects model indicated a highly positive link of 0.429 (95% CI [0.342, 0.501]) between PTG and social support. The association between PTG and social support was steady, as demonstrated by the Z value of 9.166 and *p* < 0.001. Furthermore, the homogeneity examination for 31 single samples revealed significant heterogeneity in the selected studies (Q = 447.63; *p* < 0.001; *I*^2^ = 93.3%) and potential moderating effects.

**Table 2:** Random effects model of the relation between PTG and social support.

K	*N*	Mean *r* effect size	95% CI for *r*	Homogeneity test			Test of null (two tailed)	
				Q(*r*)	*p*	*I* ^2^	Z-Value	*p*
31	6,380	0.425	[0.342, 0.501]	447.63	0.00	93.3%	9.166	<0.001

### Publication bias

**Figure 3:**
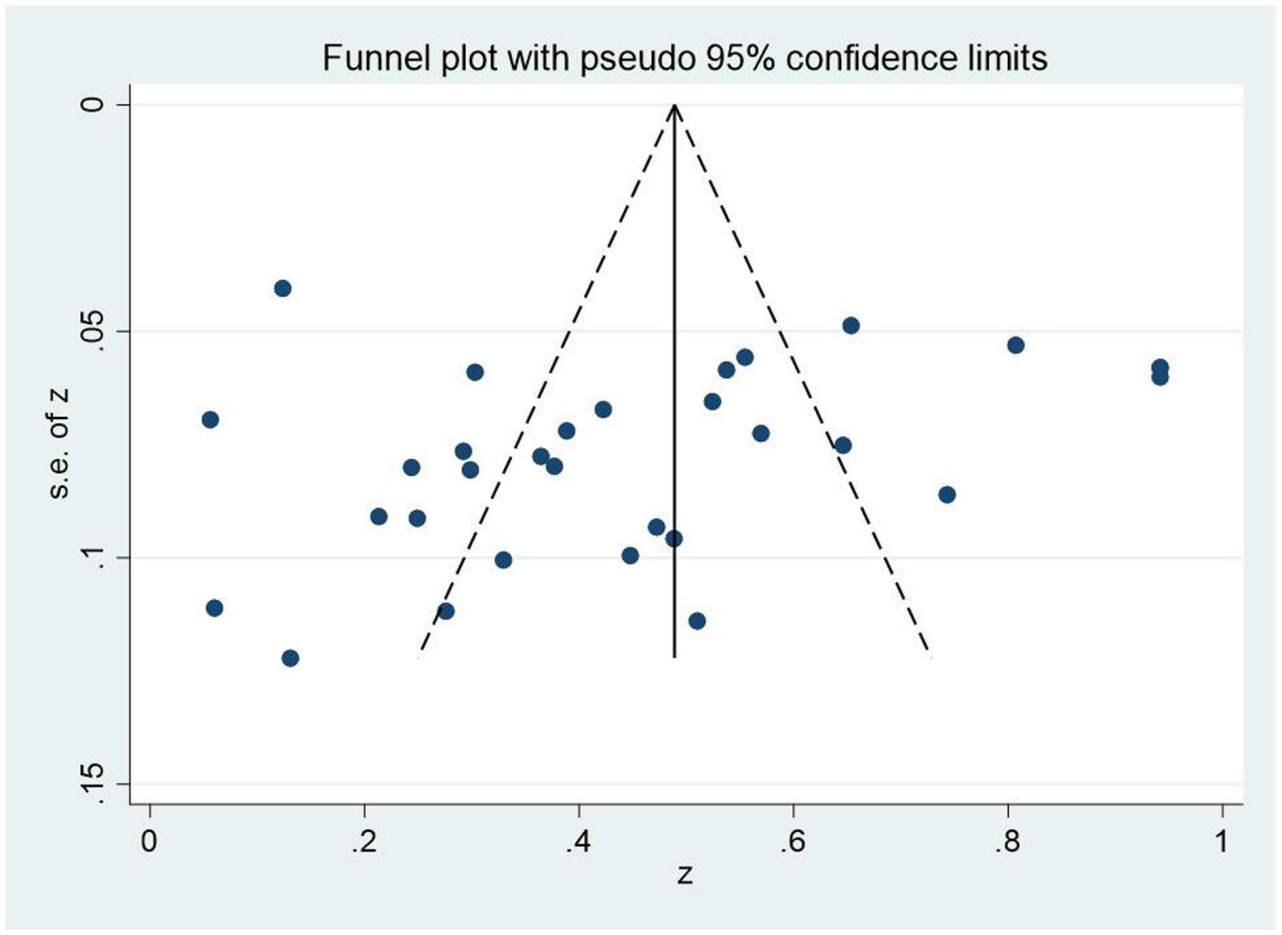
Funnel plot of the association of posttraumatic growth and social support.

First, we employed funnel plot to test whether there was publication deviations in the meta-analysis. The findings showed that the effect sizes of the correlation between PTG and social support of breast cancer patients were generally uniform distribution on two sides of the whole effect sizes (Figure 3), which meaned that there were few publication deviations. Egger’s linear regression was employed by us to test and verify this further. Egger’s linear regression test also demonstrated few significant bias (*p* = 0.229). Hence, the research population in this field could be systematically and wholly represented by the articles included in the study.

### Sensitivity analysis

We evaluate the robustness of our results by moving individual studies each time and recalculating the aggregate correlation coefficients. The sensitivity analysis results demonstrated that there had very small changes in the summary correlation coefficients between PTG and social support, indicating that our findings were steady (see Supplementary material).

### Moderating effect test

A meta-analysis of variance (Meta-ANOVA) was performed by us to examine the regulatory effect of the following target categorical variables: economic level, region, publication type, and measurement instrument for social support. In addition, a meta-regression analysis was conducted by us to verify the adjusting effects of the target continuous variables of year of publication and participants’ age.

#### Meta-ANOVA

This meta-analysis showed that economic level, region, and social support measurement tools significantly regulated the link between PTG and social support in people with breast cancer (Table 3). However, publication type (journal vs. dissertation) did not regulate the correlation between social support and PTG (Q_B_ = 1.02; *df* = 1, *p* > 0.05).

**Table 3:** PTG and social support: Univariate analysis of variance for moderators.

	Between-group effect (Q_B_)	K	N	Mean *r* effect size	95% CI for *r*	Homogeneity test within each group (Q_W_)	*I*^2^ (%)
Publication country	4.75^*^						
Developing countries		25	5,708	0.450	[0.358, 0.533]	414.88^***^	94.2
Developed countries		6	672	0.303	[0.201, 0.398]	9.86	49.3
Region	6.44^*^						
China		22	5,324	0.469	[0.371, 0.556]	394.48^***^	94.7
Foreign countries		9	1,056	0.304	[0.217, 0.385]	17.93^*^	55.4
Publication type	1.02						
Journal		24	4,718	0.400	[0.304, 0.488]	291.00^***^	92.1
Dissertation		7	2,202	0.501	[0.319, 0.647]	156.16^***^	96.2
Social support measurement	8.56^*^						
MSPSS		6	1,054	0.363	[0.212, 0.496]	32.18^***^	84.5
PSSS^a^		10	2,450	0.583	[0.478, 0.671]	118.73^***^	92.4
SSRS		7	1,376	0.408	[0.325, 0.485]	19.04^**^	68.5

a The Chinese version of PSSS revised by Jiang.

* *p* < 0.05;

** *p* < 0.01;

*** *p* < 0.001.

Economic level significantly regulated the link between PTG and social support (Q_B_ = 4.75, *df* = 1, *p* < 0.05). Specifically, the positive relation between PTG and social support was larger in developing countries (*r* = 0.450, 95% CI [0.358, 0.533]) than in developed countries (*r* = 0.303, 95% CI [0.201, 0.398]).

Region significantly regulated the correlation between PTG and social support (Q_B_ = 6.44, *df* = 1, *p* < 0.05). Specifically, the positive link between PTG and social support was larger in China (*r* = 0.469, 95% CI [0.371, 0.556]) than in foreign countries (*r* = 0.304, 95% CI [0.217, 0.385]).

Social support measurement tools significantly regulated the connection between PTG and social support (Q_B_ = 8.56, *df* = 2, *p* < 0.05). The positive link between PTG and social support was largest in the PSSS (*r* = 0.583, 95% CI [0.478, 0.671]), smaller in the SSRS (*r* = 0.408, 95% CI [0.325, 0.485]) and smallest in the MSPSS (*r* = 0.363, 95% CI [0.212, 0.496]).

#### Meta-regression analysis

We meta-regressed the *r* effect size onto the year and age in each sample to test whether the continuous variables (e.g., year and age) regulated the positive association between PTG and social support. Table 4 displays that the association between PTG and social support is not regulated by year or age.

**Table 4:** Univariate regression analysis of year and age (random effects model).

Variables	K	B	SE	95%CI	*t*	*p*
Year	31	0.007	0.012	[−0.016, 0.032]	0.65	0.521
Age	25	−0.014	0.010	[−0.043, 2.186]	−1.38	0.182

## Discussion

As far as we know, it is the first meta-analysis to explore the pooled association coefficients of PTG with social support in people with breast cancer. Our results revealed that PTG was highly positively correlated with social support in people with breast cancer, which was in accordance with the results of most research on the association between PTG and social support among people with breast cancer (Bozo et al., 2009; Yeung and Lu, 2018; Zhou, 2021). This finding supports the social support buffer hypothesis (Cohen and Mckay, 1984). It shows that good social support can play a buffering role for individuals suffering from major life events such as cancer so that patients can actively adjust their mentality to effectively cope with the disease and produce PTG. It also suggested that social support exerts an important effect in maintaining a good emotional experience for individuals (Dou et al., 2016). In addition, the results indicate that having a good social support system could help breast cancer patients with PTG. First, family support, which is reported to be significantly related to adaptation (Aass et al., 2022), especially the understanding and love of spouses, can eliminate the anxiety and sense of inferiority of breast cancer patients, enhance their sense of being respected and loved, and enable them to face life positively and overcome the disease (Bellur et al., 2018). Second, the support of friends, colleagues and medical staff can make patients feel more love and energy in their lives, thereby promoting their level of PTG (Dursun and Söylemez, 2020). Third, it is important to emphasize the role of community medical institutions and cancer nonprofit organizations, increase social attention to breast cancer patients, and improve patients’ objective support and utilization of social support.

According to the results of the Meta-ANOVA, economic level had a adjusting effect on the link between PTG and social support among people with breast cancer, and breast cancer patients in developing countries score higher than those in developed countries in the connection between PTG and social support. People with breast cancer need a variety of treatments, such as surgery, radiotherapy or chemotherapy, which may lead to disordered body image, decreased immunity, decreased self-care ability, and serious psychological issues, such as depression and anxiety (Janz et al., 2005; Tsaras et al., 2018; Davis et al., 2020). Studies have found that the social support of breast cancer patients mainly comes from medical staff, family members, and nurses (Hammersen et al., 2021; Zhang, 2021). However, compared with hospitals in developed countries, hospitals in developing countries currently have insufficient medical staff (Burmeister et al., 2019) and each nurse has a relatively high patient burden, which results in insufficient time to help solve patients’ psychological problems and provide the social support they need after completing daily treatment. This may be the reason for the discrepancies in the link between PTG and social support in people with breast cancer in developing countries and developed countries.

Additionally, region had a adjusting effect on the correlation between PTG and social support among people with breast cancer, and breast cancer patients in China score higher than those in foreign countries in the relationship between PTG and social support. This result is consistent with our previous hypothesis. The reason for this difference may also be that most of the articles included in our research were from China, and the number of Chinese articles differs greatly from that of foreign articles. Thus, more empirical studies are needed in the future to test and verify our outcomes.

Unpublished studies should be included in meta-analyses to decrease publication deviations (Sterne et al., 2000). However, although the effect sizes of journals and dissertations were different in the literature we included in this meta-analysis, this discrepancy was not noticeable; that is, the quality of the studies on the link between PTG and social support was relatively stable. Given the results of the publication bias examination, this study is less likely to have publication bias, which is consistent with the results of the moderation effect test for publication type. At the same time, considering that this study included 7 master’s and doctoral dissertations which were not officially published in journals, this also reflects the importance of the publication bias test; that is, we need to be cautious when citing the research of others. If only published journal literature is included in a meta-analysis, the representativeness of the research results will be weakened.

According to the results of the meta-regression, the year of publication did not regulate the positive connection between social support and PTG. The reason for this phenomenon may be, first, that most of the research in our study were released in the past 10 years, which is a small time span. Second, the distribution of the number of studies varied little from year to year, which may restrict the findings. Third, although the incidence of breast cancer patients is increasing yearly, with the improvement of the medical service system, people with breast cancer have a rich source of social support (Sørensen et al., 2020), which can help them grow after trauma. We found that participants’ age did not moderate the link between social support and PTG, which is different from the results of the study result of Boyle et al. (2017). This may be because breast cancer is not a single event but consists of multiple chronic traumas (Wan et al., 2022). For patients of different ages, the cause of trauma may be the diagnosis of cancer or difficult cancer treatment (Tomita et al., 2017). These different kinds of trauma lead patients to seek help from medical staff, as a result, the connection between these two variables changes little.

The social support measurement tools significantly adjusted the association between PTG and social support among people with breast cancer. We found the positive connection between social support and PTG was largest when using the PSSS, smaller when using the SSRS, and smallest when using the MSPSS. First, the reason for the difference between the MSPSS and the SSRS may be that the theoretical basis and dimensions of the two social support measurement tools are different, as is the number of measurement questions (Zimet et al., 1988; Xiao, 1994). Second, the reason for the difference between the PSSS and the MSPSS may be that the cultural backgrounds of the two scales and the content of the items are different (Jiang, 1999). In addition, to ensure the accuracy and solidity of the results, subgroups with less than 5 effect sizes were not included in the subgroup analysis (Card, 2012). Therefore, whether the link between PTG and social support is affected by the use of a smaller number of individual testing instruments remains to be confirmed in the future.

### Limitations and prospects

Differing from past research on the correlation between PTG and social support among people with breast cancer, we conducted the meta-analysis method to survey the link between PTG and social support among people with breast cancer, clearing the dispute about the extent and degree of the association between them. But this study has some limitations. First, to minimize the potential source of heterogeneity, we only chose the studies of PTG measurement instruments measured by a revised PTGI scale or the PTGI. As a result, the studies involved in our meta-analysis were limited; therefore, attention should be given to the interpretation of the results, which might have been underpowered. In addition, we only performed moderating effect analysis on the variables of economic level, region, publication type, publication year, participants’ age, and social support measurement tools. Whether variables such as, time since diagnosis, disease stage, and disease treatment moderate the link between PTG and social support among people with breast cancer can be further investigated in the future.

## Conclusion

Although this study has some limitations, all available evidence suggests a highly positive connection between PTG and social support among people with breast cancer. The summary Pearson’s correlation coefficient was 0.429. This means that people with breast cancer with high degrees of social support were more likely to have a high level of PTG. Economic level, region, and social support measurement tools adjusted the positive connection between social support and PTG, while publication type, year of publication, and participants’ age did not play a role in regulating either. Whether variables such as time since diagnosis, disease stage, and disease treatment moderate the connection between PTG and social support among people with breast cancer can be further investigated in the future.

## Author contributions

XM and XW conceived and designed the study, developed the search strategy, did the literature search, contributed to data acquisition and analysis, and contributed to writing of original manuscript. XM and CC were responsible for the software and were responsible for revising and reviewing. All authors contributed to the article and approved the submitted version.

## Funding

The work was supported by the Humanities and Social Sciences youth project of Liaoning Provincial Department of Education (WQ2020012) and Henan Graduate Education Reform and Quality Improvement Project (Grant No. YJS2021AL074).

## Conflict of interest

The authors declare that the research was conducted in the absence of any commercial or financial relationships that could be construed as a potential conflict of interest.

## Publisher’s note

All claims expressed in this article are solely those of the authors and do not necessarily represent those of their affiliated organizations, or those of the publisher, the editors and the reviewers. Any product that may be evaluated in this article, or claim that may be made by its manufacturer, is not guaranteed or endorsed by the publisher.
