# Corrigendum: The correlation between posttraumatic growth and social support in people with breast cancer: A meta-analysis

**DOI:** 10.3389/fpsyg.2023.1129481

**Published:** 2023-02-14

**Authors:** Xiaojing Ma, Xiao Wan, Chaoran Chen

**Affiliations:** Institute of Nursing and Health, College of Nursing and Health, Henan University, Kaifeng, Henan, China

**Keywords:** breast cancer, posttraumatic growth, social support, meta-analysis, review

In the published article, there was an error in the Funding statement. This work was not supported by the Humanities and Social Sciences youth project of Liaoning Provincial Department of Education (WQ2020012). The correct Funding statement appears below.

