# Supplementary material for: The correlation between posttraumatic growth and social support in people with breast cancer: A meta-analysis
Source: Front Psychol. 2022 Dec 15;13:1060150. doi: 10.3389/fpsyg.2022.1060150 (PMC9799164; doi:10.3389/fpsyg.2022.1060150)

The search strategy in PubMed

- #1 Search: "Breast Neoplasms"[Mesh] Sort by: Most Recent
- #2 Search: ((((((((((((((((((((((((((((((((((((Breast Neoplasm[Title/Abstract]) OR (Neoplasm, Breast[Title/Abstract])) OR (Neoplasms, Breast[Title/Abstract])) OR (Breast Tumor[Title/Abstract])) OR (Breast Tumors[Title/Abstract])) OR (Tumor, Breast[Title/Abstract])) OR (Tumors, Breast[Title/Abstract])) OR (Breast Cancer[Title/Abstract])) OR (Cancer, Breast[Title/Abstract])) OR (Mammary Cancer[Title/Abstract])) OR (Mammary Cancers[Title/Abstract])) OR (Cancer, Mammary[Title/Abstract])) OR (Cancers, Mammary[Title/Abstract])) OR (Malignant Neoplasm of Breast[Title/Abstract])) OR (Breast Malignant Neoplasm[Title/Abstract])) OR (Breast Malignant Neoplasms[Title/Abstract])) OR (Malignant Tumor of Breast[Title/Abstract])) OR (Breast Malignant Tumor[Title/Abstract])) OR (Breast Malignant Tumors[Title/Abstract])) OR (Cancer of Breast[Title/Abstract])) OR (Cancer of the Breast[Title/Abstract])) OR (Mammary Carcinoma, Human[Title/Abstract])) OR (Mammary Carcinomas, Human[Title/Abstract])) OR (Carcinoma, Human Mammary[Title/Abstract])) OR (Carcinomas, Human Mammary[Title/Abstract])) OR (Human Mammary Carcinoma[Title/Abstract])) OR (Human Mammary Carcinomas[Title/Abstract])) OR (Mammary Neoplasm, Human[Title/Abstract])) OR (Mammary Neoplasms, Human[Title/Abstract])) OR (Human Mammary Neoplasm[Title/Abstract])) OR (Human Mammary Neoplasms[Title/Abstract])) OR (Neoplasm, Human Mammary[Title/Abstract])) OR (Neoplasms, Human Mammary[Title/Abstract])) OR (Breast Carcinoma[Title/Abstract])) OR (Breast Carcinomas[Title/Abstract])) OR (Carcinoma, Breast[Title/Abstract])) OR (Carcinomas, Breast[Title/Abstract])
- #3 Search: #1 OR #2
- #4 Search: "Posttraumatic Growth, Psychological"[Mesh] Sort by: Most Recent
- #5 Search: ((((((((Growth, Psychological Posttraumatic[Title/Abstract]) OR (Psychological Posttraumatic Growth[Title/Abstract])) OR (Post-traumatic Growth, Psychological[Title/Abstract])) OR (Growth, Psychological Post-traumatic[Title/Abstract])) OR (Post traumatic Growth, Psychological[Title/Abstract])) OR (Psychological Post-traumatic Growth[Title/Abstract])) OR (Psychological Post-traumatic Growths[Title/Abstract])) OR (Posttraumatic Growth[Title/Abstract])) OR (Growth, Posttraumatic[Title/Abstract])
- #6 Search: #4 OR #5
- #7 Search: " Social Support"[Mesh] Sort by: Most Recent
- #8 Search: (((((((((((((support, social[Title/Abstract]) OR (social care[Title/Abstract])) OR (care, social[Title/Abstract])) OR (online social support[Title/Abstract])) OR (online social supports[Title/Abstract])) OR (social support, online[Title/Abstract])) OR (social supports, online[Title/Abstract])) OR (support, online social[Title/Abstract])) OR (perceived social support[Title/Abstract])) OR (perceived social supports[Title/Abstract])) OR (social support, perceived[Title/Abstract])) OR (social supports, perceived[Title/Abstract])) OR (support, perceived social[Title/Abstract])) OR (supports, perceived social[Title/Abstract])
- #9 Search: #7 OR #8
- #10 Search: #3 AND #6 AND #9

JBI Critical Appraisal Checklist for Studies Reporting Prevalence Data

Items	Yes	No	Unclear	Not applicable
1. Was the sample frame appropriate to address the target population?				
2. Were study participants sampled in an appropriate way?				
3. Was the sample size adequate?				
4. Were the study subjects and the setting described in detail?				
5. Was the data analysis conducted with sufficient coverage of the identified sample?				
6. Were valid methods used for the identification of the condition?				
7. Was the condition measured in a standard, reliable way for all participants?				
8. Was there appropriate statistical analysis?				
9. Was the response rate adequate, and if not, was the low response rate managed appropriately?				

Sensitivity analysis of the 31 studies included in the meta-analysis

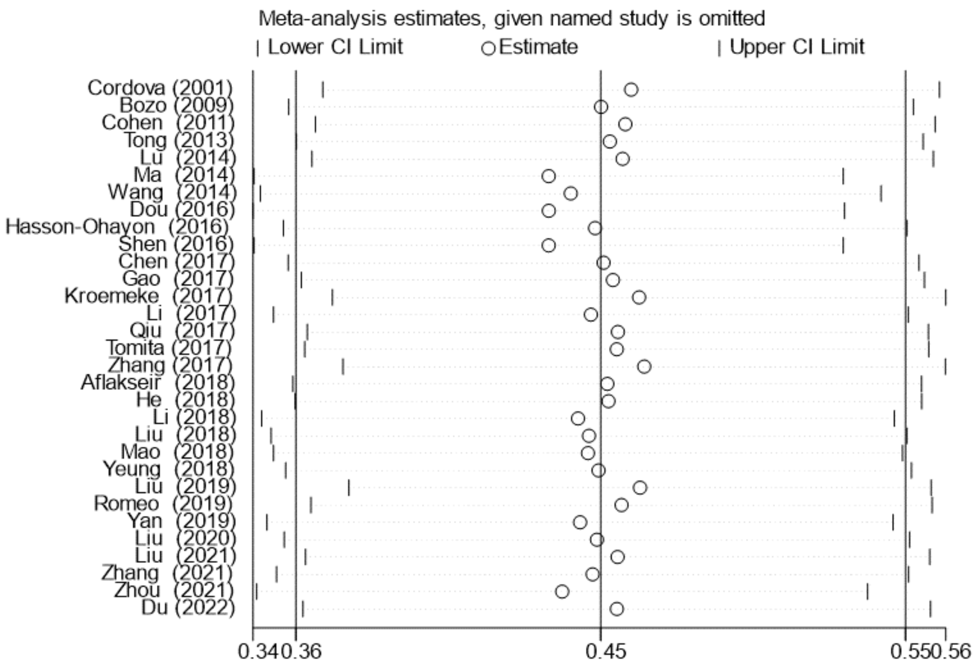

Supplement: Supplementary file 1 [file Data_Sheet_1.PDF]
